# Development of cardiorespiratory fitness standards for working memory using receiver operating curves in 15-year-old adolescents

**DOI:** 10.1186/s12887-021-02681-5

**Published:** 2021-04-29

**Authors:** Vinícius Muller Reis Weber, Daniel Zanardini Fernandes, Leonardo Alex Volpato, Maria Raquel de Oliveira Bueno, Marcelo Romanzini, Jose Castro-Piñero, Enio Ricardo Vaz Ronque

**Affiliations:** 1 Laboratory of Physical Activity and Health, Center of Physical Education and Sports, Londrina State University - UEL, Londrina, Paraná Brazil; 2 GALENO Research Group Department of Physical Education, School of Education, University of Cadiz, Puerto Real, Spain; 3 Biomedical Research and Innovation Institute of Cadiz (INiBICA) Research Unit, 11009 Cadiz, Spain

**Keywords:** Executive functions, Cognitive Control, Physical Fitness, Development

## Abstract

**Background:**

Working memory performance is associated with better academic achievements in children and adolescents, and it is positively related to CRF. However, what level of cardiorespiratory fitness (CRF) discriminates higher working memory performance is not known. The purpose of this study was to identify CRF thresholds linked to working memory in adolescents.

**Methods:**

Data of 141 adolescents (53.2 % girls) were collected (14.9 years) from a cross-sectional study during the year 2019. CRF was assessed by the 20-m shuttle run test, and maximal oxygen uptake was calculated using the Mahar´s equation. Working memory was evaluated by the Corsi blocks test and performance was classified by percentiles. Receiver operating characteristic (ROC) curve analysis was used to identify CRF thresholds.

**Results:**

The ROC analysis indicated that CRF could be used to discriminate working memory in adolescents. CRF thresholds of ≥45.03 ml.kg^− 1^.min^− 1^for boys and ≥36.63 ml.kg^− 1^.min^− 1^for girls were found to be indicative of “normal” working memory performance.

**Conclusions:**

CRF could discriminate low and normal working memory performance in 14-16- year-old adolescents. These thresholds could allow for earlier identification and intervention of low working memory performance using CRF.

## Background

Cardiorespiratory fitness (CRF) and physical activity (PA) have shown positive effects on young people’s cognition [1]. Several systematic reviews have suggested that CRF and PA promote benefits in academic [2, 3] and cognitive performance [1, 3, 4] of children and adolescents. In this way, PA has the potential to improve or maintain CRF, which in turn can affect brain plasticity [5], leading to improvements in both academic performance and executive functions [6].

Well-developed executive functions are necessary requirements for good academic performance [7]. Among executive functions, working memory stands out, which is a highly important function in the learning and academic performance of children and adolescents [8]. Working memory is responsible for monitoring and coding the information received in order to review and replace information that is no longer relevant due to new and more useful information [7]. Moreover, for the formation of long-term memory, necessary for the learning process, the information must firstly be encoded as working memory; the construction of new concepts is a process of joining different items together, and these items were firstly kept in mind by the working memory process [9].

Working memory has been associated with CRF [10, 11] in children and adolescents, and it has been suggested that PA promotes improvements in physical fitness and improvements in brain structures that support executive functions and memory [1, 11]. Bruijn et al. [11], found that among executive functions analyzed, visuospatial working memory mediated associations between academic achievements and physical fitness. Hansen et al. [12] observed that CRF had significant quadratic association with academic performance (spelling and mathematics), indicating that 22 to 27 PACER laps were key to significant increases in academic performance of children.

It is known that CRF is used as an important discriminator for health factors in young people [13, 14]. Studies have used CRF levels to discriminate metabolic syndrome [15] and cardiovascular health [16] in adolescents. In addition, many studies have demonstrated the importance of CRF for executive functions in children. These studies have created groups of high and low maximal oxygen uptake (VO_2max_),in children by the percentile, and compared these groups for performance in cognitive functions [17–20]; and most of these studies excluded individuals classified in middle percentiles. However, little is known about how much CRF is necessary to be classified with good working memory in adolescence, since most studies have been carried out with children, and information for adolescents is scarce [21].

Furthermore, working memory performance increases over the years, and it increases with childhood maturation; in this sense, adolescents are capable of retaining more information than children [9]. In addition, the VO_2max_ value necessary to stimulate the working memory performance in adolescents is not yet clear.

In this case, a specific threshold could provide the ideal cutoff for a better performance in working memory for adolescents. Therefore, the objective of the present study was to create cutoff CRF points in order to discriminate the working memory performance using receiver operating characteristic (ROC) analysis in adolescents.

## Methods

### Sample and study design

This was a cross-sectional study involving students from public schools of Londrina-PR, Brazil. The sample was composed of 141 adolescents (75 girls), aged 14.9 years and enrolled in secondary education. Adolescents who did not return the consent form signed by parents/guardians and declared withdrawal during or after data collection were excluded from the study.

The data collection process included obtaining anthropometric measurements, CRF, and working memory. Measurements were performed on two days on the school settings. Working memory and CRF tests were applied on different days to avoid possible interferences. Data were collected during the year 2019.

### Anthropometric measurements

Body mass was measured using portable digital scale, with precision of 0.1 kg (Seca, Hamburg, Germany) and height with portable stadiometer, with precision of 0.1 cm (Harpenden Holtain Ltd, Crymych, Dyfed, UK). From this, Body Mass Index (BMI) was estimated (kg/m^2^). Sum of skinfolds was collected through subscapular and tricipital skinfold thickness, which were measured using scientific adipometer (Lange, Cambridge Scientific Instruments, Cambridge, MD) and performed by experienced evaluator in accordance to techniques stablished by Harrison et al. [22].The absolute technical error was 0.4 cm for height and 1.3mm for tricipital and 0.96mm for subscapular skinfolds.

### Cardiorespiratory Fitness

CRF was evaluated by the 20-m shuttle-run test. This test was conducted on a sports court and the criteria for conduction and completing the test followed procedures described by Léger et al. [23]. The test started at velocity of 8.5 km/h and had increment of 0.5 km/h every minute. The end of the test was determined by voluntary exhaustion or failure to maintain the velocity determined by each stage in three consecutive signals. VO_2max_ was calculated in ml.kg^− 1^.min^− 1^, using the quadratic equation suggested by Mahar et al. [24], and recommended by the FITNESSGRAM.

### Working memory

To verify the working memory, the Corsi block-tapping task (CB) was used. The Corsi Block Test is widely used both in clinical practice and research, and specifically evaluates short-term visuospatial working memory [25]. Originally developed by Corsi [26], this test involves simple measurements that can be quickly and easily administered, requiring the subject to maintain the information sequence [25]. This test have good reliability for 15-year-old adolescents (*r* = 0.79) and moderate validity (*r* = 0.66) [27].

According to the normative standardization of the Corsi block test, 20 % of individuals with the worst test results can be considered as “low performance” [25]. In addition, subjects classified with 0.6 z-score below the group that they belong are identified with low neuropsychological ability. In the same way, if the test is easy, 80 % could be classified as middle score [28].

**Fig. 1:**
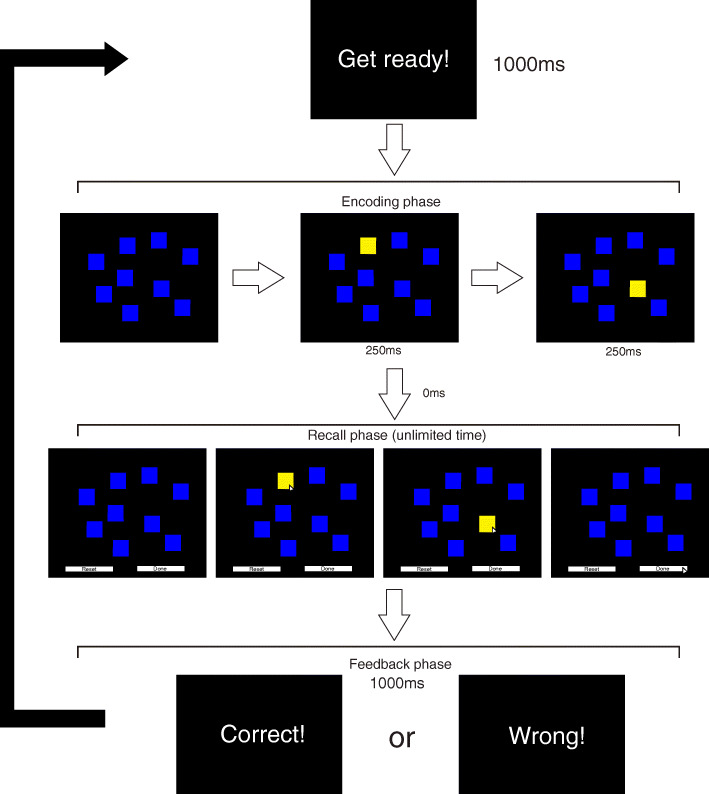
Corsi block task

The test consists in memorizing a sequence in which cubes flash on a computer screen. The test starts with two cubes flashing in the middle of nine cubes disposed on the screen (encoding phase), each cube flashing for 250ms. In sequence, the adolescent tries to reproduce the sequence in the same order (forward condition) in which cubes appears (recall phase), which does not have time to finish. After the response, the adolescent received feedback (1000ms) and start a new encoding phase (Fig. 1). The order in which cubes flash increases progressively until maximum limit is reached. The test was interrupted when the participant misses the sequence order twice at the same level. Block Span (CB extension) and total score (block span x number of correct responses until the test was interrupted) were adopted as performance indicators. Adolescents had one execution in the test for adaptation.

For classification into normal and low performance, 20 percentiles as described by Kessels et al. [25] was used. Adolescents who obtained the 20 % lowest scores were classified as low performance. Classification was performed according to sex.

### Statistical analyses

Median and interquartile ranges were used for sample description. Mann Whitney U was adopted for comparison of variables between groups. ROC analysis was used to discriminate adolescents with normal performance from those with low performance for working memory with VO_2max_. For ROC analysis, the area under the curve (AUC) was used for analysis of the accuracy of cutoff points. Better ROC analysis results are found for sensitivity and specificity close to 100, in this case indicating high positive cases and low false-positive cases.

After identifying cutoff values, two groups were created, high CRF (Boys≥45.03; Girls≥36.63) and Low CRF (Boys < 45.03; Girls < 36.63) in order to compare groups; the comparison was performed by the generalized estimating equation and values were expressed in estimated means and confidence intervals. Analyses were controlled by sex and ∑ of tricipital and subscapular skinfolds. Significance was set at 5 %. All analyses were conducted using statistical software SPSS version 26.0 and MedCalc version 19.1.2.

## Results

Table 1 shows the sample characterization between subjects classified as normal or low performance and stratified by sex. Results demonstrate significant differences for VO_2max_ (Girls: *P* = 0.023; Boys: *P* = 0.037) and for working memory variables (Block Span and Total Score, *P* < 0.001). Higher CRF values and working memory performance were found for adolescents classified as normal cognitive performance.

**Table 1:** Characterization of the sample stratified by sexes and working memory performance

	Girls (n = 75)			Boys (n = 66)			
	**Low**	**Normal**	***P***	**Low**	**Normal**	***P***	
**Age (years)**	15.01 (14.54–15.19)	14.96 (14.54–15.42)	0.822	14.82 (14.09–15.99)	15.05 (14.55–15.47)	0.587	
**Body Mass (Kg)**	55.9 (53.0–67.65)	55.25 (50.92–65.95)	0.455	64.80 (53.15–82.35)	59.6 (54.75–69.8)	0.232	
**Height (cm)**	161.5 (156.85–169.55)	163.55 (158.12–168.52)	0.728	172.4 (164.5–176.15)	171.7 (167.7–175.65)		0.764
**BMI (Kg/m²)**	21.85 (20.29–25.56)	21.15 (19.09–24.97)	0.351	22.13 (17.55–27.47)	20.35 (18.20–22.70)	0.210	
**∑ skinfold (mm)**	42 (32.50–59.5)	39.75 (31.0–54.5)	0.441	37.0 (18.25–51.0)	26.0 (19.0–37.75)	0.278	
**Vo**_**2max**_**(ml.kg**^**− 1**^.**min**^**− 1**^**)**	35.71 (33.19–39.60)	40.01 (34.19–42.82)	0.023	47.39 (39.38–52.52)	50.09 (46.79–54.28)	0.037	
**Corsi Blocks** Block Span Total Score	5 (5–5) 35 (27.5–35)	6 (5–7) 54.0 (40–70)	< 0.001 < 0.001	5 (5–5) 35 (30–35)	6 (6–7) 54 (45–70)	< 0.001 < 0.001	

*BMI* Body mass index;* ∑ skinfold*: sum of skinfold; Values expressed in median and interquartile range; Significant values for *P* < 0.05

**Fig. 2:**
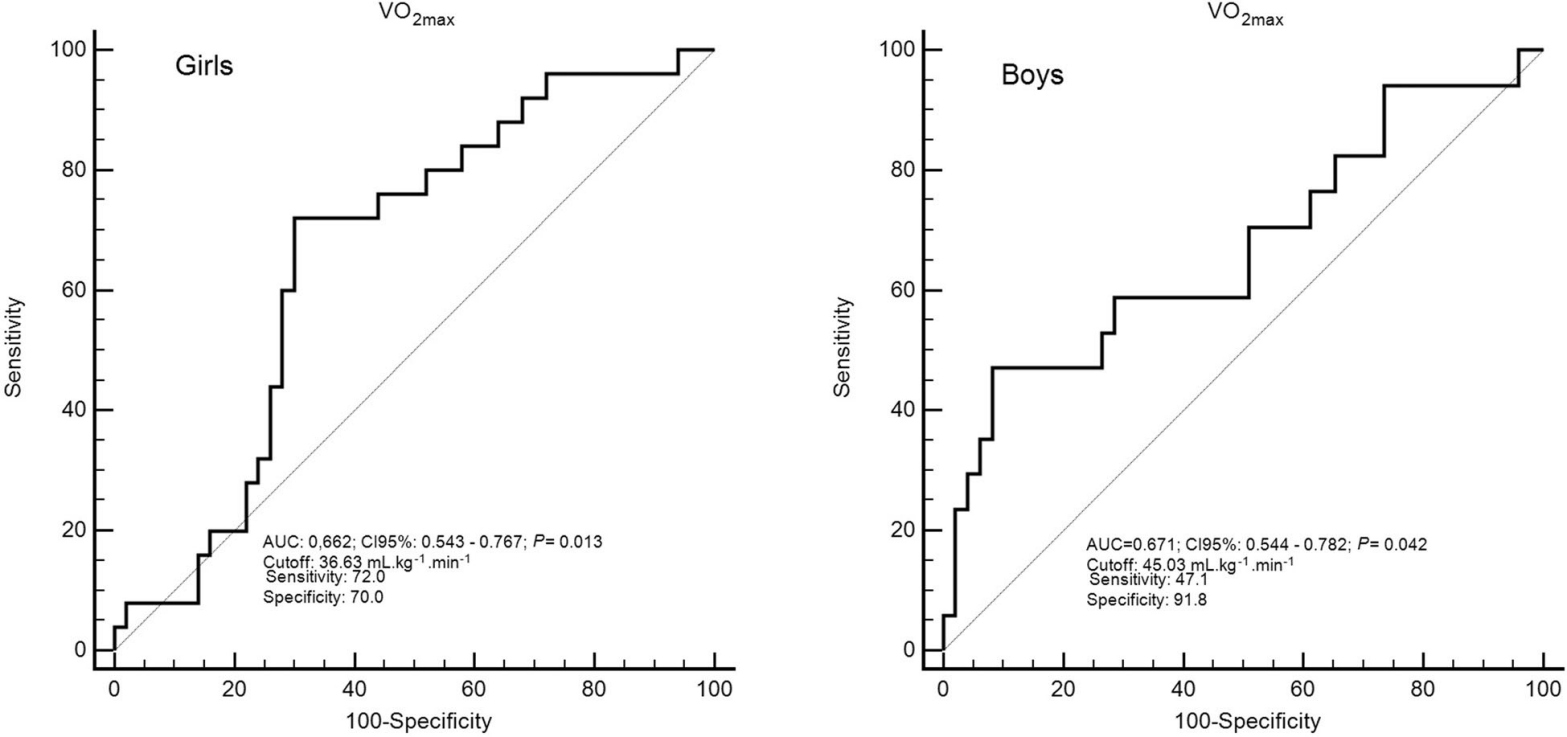
Receiver operating characteristic curves Note: AUC: area under the curve

Figure 2 presents the ROC Curves analysis for VO_2max_ and working memory classification (low or normal). In both sexes, VO_2max_ was able to discriminate subjects with low and normal working memory ability (*P* < 0.05). For boys, the cutoff value to be classified as “normal” was ≥45.03 ml.kg^− 1^.min^− 1^ (Sensitivity 47.1; Specificity 91.8); VO_2max_ was able to discriminate cases with accuracy of 67.1 % (AUC = 0.671; CI95 %: 0.544–0.782). For girls, the value was ≥36.63 ml.kg^− 1^.min^− 1^ (Sensitivity 72; Specificity 70) and discriminate normal or low performance subjects with accuracy of 66.2 % (AUC = 0.662; CI95 %: 0.543–0.767).

Comparisons between high and low CRF are shown in Table 2. Significant differences were found for VO_2max_ and total score in Corsi Blocks test for all boys, demonstrating that boys classified as high CRF presented higher working memory values if compared to low CRF individuals (df:16.22; *P* = 0.001).

**Table 2:** – Comparison of cardiorespiratory fitness groups for VO_2max_ and working memory performance in adolescents

	N	VO_2max_ (ml.kg^− 1^.min^− 1^) Estimated means (95 % CI)	*P*	Total Score Estimated means (95 % CI)	*P*
**All** Low CRF High CRF	45 96	34.3 (33.0–35.7) 47.1 (46.0–48.3)	< 0.001	43.7 (38.7–49.4) 53.2 (49.5–57.35)	0.005
**Boys** Low CRF High CRF	12 54	39.5 (37.5–41.5) 51.1 (50.2–52.1)	< 0.001	39.5 (32.5–47.9) 55.7 (50.6–61.3)	0.001
**Girls** Low CRF High CRF	33 42	32.4 (31.2–33.6) 41.9 (41.0–42.9)	< 0.001	45.3 (39.1–52.4) 50.1 (44.7–56.1)	0.280

*CRF* Cardiorespiratory fitness; *95 % CI* Confidence interval of 95 %; Significant values for *P* < 0.05

## Discussion

The main objective of the present study was to verify the level of CRF (VO_2max_) that discriminates working memory in adolescents. Results showed that boys need minimum of 45.03 ml.kg^− 1^.min^− 1^ and girls 36.63 ml.kg^− 1^.min^− 1^ to be classified as “normal”. Additionally, VO_2max_ thresholds presented significant differences for working memory, which demonstrated higher working memory values for group with high CRF.

Comparing CRF groups with academic achievement, adolescents classified in the Health Fitness Zone (HFZ), FITNESSGRAM^®^, presented higher academic grades [1, 29]. When the relationship between CRF and academic achievement in children was evaluated, Hansen et al. [12] found non-linear relationship, with increases in academic performance occurring up to 22 laps (~ 47.5ml.kg^− 1^.min^− 1^) for spelling and 27 (~ 49.7 ml.kg^− 1^.min^− 1^) laps for math scores, after that, performance reached a plateau.

The relationship between CRF (pacer laps) and working memory performance, in children, presented significance, analyzing the reaction time (r= -0.13), and working memory accuracy (r = 0.14) [30]. Analyzing relationships longitudinally and controlling other variables (grade, sex, maternal education, BMI), CRF can explain by 7.5 % the working memory accuracy [31]. In addition, improvements in CRF are associated with improvements in the cognitive control of the working memory of preadolescents [32].

According to results of the present study, higher CRF values, classified by the created threshold, indicated better working memory for boys. However, for girls, this result did not show significance; a possible justification to this result is that the maturation of the cognitive control takes longer for boys than for girls [33]. Thus, girls probably suffer less CRF impact on cerebral structures. At the age of 15 years, girls have some advantages (cortical thickness) in brain regions [34], responsible for the working memory. Haapala et al. [35] found significant positive relationship for physical activity and academic achievement for boys and not for girls, suggesting differences between boys and girls. These differences, mainly related to cognitive functions, are not well understood, and further studies are needed.

The threshold values for VO_2max_ to discriminate low and normal working memory in the present study are similar to those used by FITNESSGRAM®, developed by Welk et al. [15], which determined the presence of metabolic syndrome through VO_2max_ measured by submaximal treadmill test. For boys, the HFZ value was ≥43.6 ml.kg^− 1^.min^− 1^, a difference of 1.7 ml.kg^− 1^.min^− 1^ from the present study; for girls, the HFZ value was≥39.1 ml.kg^− 1^.min^− 1^, showing difference of 2.47 ml.kg^− 1^.min^− 1^.

Ruiz et al. [16] established CRF cutoff points to determine the cardiovascular health profile of adolescents, finding values of 43.8 ml.kg^− 1^.min^− 1^ for boys and 34.6 ml.kg^− 1^.min^− 1^ for girls. Likewise, these values are similar to those found in the present study, demonstrating that cardiovascular health and mental functions are affected in similar CRF intensities in adolescents.

The low sensitivity and high specificity found for boys can be explained by the number of boys with high CRF in this sample. Low-performance adolescents showed median VO_2max_ value of 47.39 ml.kg^− 1^.min^− 1^ (39.38–52.52). Using the classification proposed by an international normative [36], the low-performance group had subjects in the 50th percentile but some individuals could reach the 90th percentile. On the other hand, the high specificity of created cutoff points (91.8 %) implies that individuals with low CRF are more likely of being classified as low performance. This pattern was not found for girls, in which the similar sensitivity and specificity could be justified by the homogeneous VO_2max_ distribution.

Another fact to be highlighted is the changes that CRF can cause in cerebral morphology, and these changes are related to working memory performance. It is noteworthy that well-developed prefrontal cortex [37, 38] and greater hippocampal volume [39] are associated with better working memory. In addition, children with higher CRF have greater hippocampal volume [39, 40]. The relationship between CRF and relational memory is mediated by the hippocampal volume [39]. Better working memory is related to joining different concepts to solve a problem that may require a combination of different strategies, and more complex tasks require the joining of more parts [9].

Neuroelectric indexes are also related to working memory, and evidence suggests that higher P3 amplitude, an event related to the neuronal activity and linked to attentional processing, is associated with better working memory [6, 41]. Comparing to individuals with high and low CRF by Event-Related Potentials, the results demonstrated better P3 indexes for children with high CRF [10, 42]; and higher functional connectivity [20]. The increase in neuronal connectivity, resulting from neuronal density and myelination, mainly in areas of the brain responsible for the working memory, could lead to better working memory performance [43].

Further studies could assess the possible cause and effect relationship between CRF and working memory performance. Further studies should also evaluate CRF by aerobic training and its relationship with working memory; therefore, more randomized controlled trials are necessary to elucidate this relationship. Last but not least, new studies are required to evaluate sex differences associated to CRF impacts on cognitive performance.

This study has some limitations such as the relatively small sample size and the creation of thresholds only for adolescents aged 14–16 years. Another limitation was not using academic achievements for the development of thresholds to compare working memory. However, a strong point is the creation of thresholds for working memory in adolescents, which is possibly one of the first studies with this objective. In addition, this study used sample composed of adolescents, and many studies used children in the second infancy [21].

## Conclusions

In summary, VO_2max_ can be used to discriminate adolescents classified as low or normal working memory performance. In this sense, these results can complement normative health data or be useful in school programs, since working memory can improve academic performance.

## Data Availability

The datasets used and/or analyzed during the current study are available from the corresponding author on reasonable request.

## Abbreviations

CRF: Cardiorespiratory fitness
PA: Physical activity
VO_2max_: Maximal oxygen uptake
ROC: Receiver operating characteristic
BMI: Body mass index
AUC: Area under curve
HFZ: Health fitness zone
